# Mean Field Initialization of the Annealed Importance Sampling Algorithm for an Efficient Evaluation of the Partition Function Using Restricted Boltzmann Machines

**DOI:** 10.3390/e27020171

**Published:** 2025-02-06

**Authors:** Arnau Prat Pou, Enrique Romero, Jordi Martí, Ferran Mazzanti

**Affiliations:** 1 Departament de Física, Universitat Politècnica de Catalunya, Barcelona Tech, Campus Nord B4-B5, E-08034 Barcelona, Spain; arnau.prat.pou@upc.edu (A.P.P.); jordi.marti@upc.edu (J.M.); 2 Departament de Ciències de la Computació, Universitat Politècnica de Catalunya, Barcelona Tech, Campus Nord B4-B5, E-08034 Barcelona, Spain; eromero@cs.upc.edu

**Keywords:** magnetic systems, partition function, annealed importance sampling, Restricted Boltzmann Machines

## Abstract

Probabilistic models in physics often require the evaluation of normalized Boltzmann factors, which in turn implies the computation of the partition function *Z*. Obtaining the exact value of *Z*, though, becomes a forbiddingly expensive task as the system size increases. A possible way to tackle this problem is to use the Annealed Importance Sampling (AIS) algorithm, which provides a tool to stochastically estimate the partition function of the system. The nature of AIS allows for an efficient and parallel implementation in Restricted Boltzmann Machines (RBMs). In this work, we evaluate the partition function of magnetic spin and spin-like systems mapped into RBMs using AIS. So far, the standard application of the AIS algorithm starts from the uniform probability distribution and uses a large number of Monte Carlo steps to obtain reliable estimations of *Z* following an annealing process. We show that both the quality of the estimation and the cost of the computation can be significantly improved by using a properly selected mean-field starting probability distribution. We perform a systematic analysis of AIS in both small- and large-sized problems, and compare the results to exact values in problems where these are known. As a result, we propose two successful strategies that work well in all the problems analyzed. We conclude that these are good starting points to estimate the partition function with AIS with a relatively low computational cost. The procedures presented are not linked to any learning process, and therefore do not require a priori knowledge of a training dataset.

## 1. Introduction

The evaluation of thermodynamic potentials such as the entropy or free energy is key to understanding the equilibrium properties of physical systems [1]. In real-sized classical problems, computer simulations based on Molecular Dynamics or Monte Carlo methods cannot generically access them, mainly because of the size of the space of states to sample, which grows exponentially with the number of particles. This effect is particularly easy to quantify in magnetic models of classical two-state spin systems, where the volume of the phase space grows as $2^{N}$, with *N* the total number of spins. Quantities such as the Helmholtz free energy *F* in the canonical ensemble, proportional to the logarithm of the partition function [2,3]

(1) $$Z=\sum_{x}e^{-E\left(x\right)/k_{B}T},$$

are out of reach, as the sum extends over all possible states x, with E(x) the corresponding energy, $k_{B}$ the Boltzmann constant, and *T* the temperature. Actually, finding the value of *Z* is known to be an NP-hard problem [4] that therefore prevents an exact estimation unless the system is small.

The relevance but unfortunate computational complexity implied in the determination of *Z* has raised the urge to devise methods to approximate it in a tractable way. One remarkable technique designed to tackle this problem was developed by Bennett [5], where the free energy difference between two overlapping canonical ensembles is estimated directly in a Monte Carlo simulation. In case one of the two values of *F* is known, the method allows us to obtain the value of the other, thus gaining access to $F=-k_{B}T\log\left(Z\right)$. Another interesting approach towards the evaluation of the partition function is derived from the Wang–Landau algorithm [6,7,8], where a stochastic exploration of the phase space is used to recover the density of energy states ρ(E) corresponding to the Hamiltonian of the system under study. In this framework, the partition function is recovered as the integral of $\rho \left(E\right)e^{-E/k_{B}T}$ over the energy range spanned by the system configurations. This method has proved to reliably reproduce the physics of different systems such as the 2D-Ising model, although it can be difficult to apply to more complex situations involving an intricate ρ(E).

An alternative approach to the problem was devised in 2001 by R. M. Neal [9,10], the Annealed Importance Sampling algorithm, where an annealing procedure is implemented to obtain reliable samples from an otherwise intractable probability distribution starting from samples of a simpler and tractable one. In this method, the partition function is one of the simplest quantities to evaluate, although as in most sampling schemes, convergence towards the exact value of *Z* is only guaranteed in the infinite limit, both in number of samples and intermediate annealing steps. In practical terms, when a finite number of samples and intermediate annealing chains is employed, the predicted value of *Z* depends on the different simulation inputs, particularly on the initial probability distribution.

Surprisingly, and despite its broad formulation in terms of an initial and a final probability distribution, little use has been seen of the AIS algorithm in the numerical simulation of physical systems, to the best of our knowledge. More applications have emerged in the world of neural networks, particularly in the field of machine learning with RBMs [11,12], where the evaluation of *Z* is key to a precise optimization of the system parameters along learning in an exact gradient descent scheme. In this context, the AIS algorithm turns out to be most efficient since the random walk exploration can be performed by means of Gibbs sampling, which is fully parallelizable [13]. A review and unifying framework of the algorithms for the estimation of the partition function with AIS in RBMs can be found in [14].

In any case, the AIS algorithm is particularly suited to addressing binary state unit problems like spin systems or RBMs where the different probability distributions involved along the annealing chains are cost-effective and simple to evaluate. Notice that the RBM is a mathematical model that can be used to describe magnetic spin systems, where the weights and bias are directly related to the correlations, external fields and temperature (usually known or modeled a priori) [15,16,17]. In this sense, an RBM can be used to analyze the thermodynamics of these systems, without resorting to a training set or a learning scheme. In this work, we focus on that situation, as we consider the RBM network parameters to be known. We use AIS to compute the partition function of different systems at several but low temperatures, where the calculation of log(Z) is known to be harder than at T≫1. Notice, though, that AIS is a general algorithm that has a broad range of applications that go beyond its use in RBM modeling [18,19].

To be precise, in this work, we study how AIS can be used to produce reliable estimates of log(Z) in magnetic physical systems that can be mapped into RBMs. Our goal is to achieve that using a suitable starting probability distribution with a small computational cost, even in realistically large problems. We discuss how to obtain the optimal mean field probability distribution $p_{0}^{*}\left(x\right)$ that is closest to the Boltzmann distribution of the real model under study. After a brief derivation of how to obtain $p_{0}^{*}\left(x\right)$ from average system properties, we propose two strategies to find approximations to it in Ising, Spin-Glass systems and artificial models, where the exact value of the partition function can be determined. We also compare the results obtained with the standard procedure, where the uniform probability distribution is employed as the starting point of the AIS algorithm [14,20], a procedure that shows a non-stable behavior when measured along learning [21]. Notice that our methodology does not use any external data other than the two-body correlations and external fields defining the model.

## 2. Annealed Importance Sampling

The AIS algorithm, developed by R. Neal in the late 1990s [9,10], allows sampling from a probability distribution that would otherwise be intractable. It can be used to estimate *Z*, but it is more general and allows finding approximate values of any observable quantity α(x) over a probability distribution p(x). In a general sense, this computation can be very inefficient due to two main reasons. On one hand, the probability distribution p(x) can be impossible to sample because the exact form of p(x) is not known, as happens in many quantum physics problems [22,23,24,25]. On the other hand, the number of samples required to obtain an accurate estimate of the average value of α(x) may be unreasonably large. In order to deal with these problems, one usually resorts to some form of Importance Sampling, where the exploration of the space is guided by a known and suitable probability distribution q(x) [26]. In this way, one typically evaluates

(2) $$\langle \alpha \rangle =\int dx\;q\left(x\right)\left(\frac{p(x)\alpha (x)}{q(x)}\right).$$

using stochastic techniques, where samples are drawn from q(x). Importance Sampling is employed to reduce the variance of the estimator, or to reduce the number of samplings needed to achieve the same statistical accuracy. In any case, Importance Sampling can only be performed when a suitable q(x) is at hand, but that may not always be the case. The AIS method allows building q(x) starting from a trivial probability distribution, and performing an annealing process through a set of intermediate distribution corresponding to decreasing temperatures.

As explained in [9,10], in order to estimate 〈α〉 starting from a trivial $p_{0}\left(x\right)$, one builds a chain of intermediate distributions $p_{i}\left(x\right)$ that interpolate between $p_{0}\left(x\right)$ and $p_{n}\left(x\right)=p\left(x\right)$. Denoting by ${\tilde{p}}_{k}\left(x\right)=Z_{k}p_{k}\left(x\right)$ the corresponding unnormalized probability distributions, a common scheme is to define

(3) $${\tilde{p}}_{k}\left(x\right)={\tilde{p}}_{0}{\left(x\right)}^{1-\beta_{k}}{\tilde{p}}_{n}{\left(x\right)}^{\beta_{k}},$$

with $0=\beta_{0}<\beta_{1}<\cdots <\beta_{n}=1$ and $N_{\beta }=n+1$. The approach used in AIS is to turn the estimation of 〈α〉 into a multidimensional integration of the form

(4) $$\langle \alpha \rangle =\int dx_{1}\cdots dx_{n}\;g\left(x_{1},\cdots ,x_{n}\right)\frac{f(x_{1},\cdots ,x_{n})}{g(x_{1},\cdots ,x_{n})}\alpha \left(x_{n}\right),$$

where

(5) $$\begin{array}{ccc} f(x_{1},\cdots ,x_{n})\;\;\; & = & \;\;\;p_{n}\left(x_{n}\right)\prod_{j=1}^{n-1}{\hat{T}}_{j}\left(x_{j+1},x_{j}\right) \end{array}$$

(6) $$\begin{array}{ccc} g(x_{1},\cdots ,x_{n})\;\;\; & = & \;\;\;p_{0}\left(x_{1}\right)\prod_{j=1}^{n-1}T_{j}\left(x_{j},x_{j+1}\right) \end{array}$$

are normalized joint probability distributions for the set of variables $\left\{x_{1},\ldots ,x_{n}\right\}$. In these expressions, $T_{k}\left(x,y\right)$ represents a transition probability of moving from state x to state y, which asymptotically leads to the equilibrium probability $p_{k}\left(x\right)$. In the same way, ${\hat{T}}_{k}\left(y,x\right)$ represents the reversal of $T_{k}\left(x,y\right)$. The detailed balance condition implies that the transition probabilities fulfill the relation

(7) $${\hat{T}}_{j}\left(y,x\right)=T_{j}\left(x,y\right)\frac{p_{j}\left(x\right)}{p_{j}\left(y\right)}$$

in order to be able to sample the space ergodically [27]. Therefore, 〈α〉 can be estimated from Equation (4) with

(8) $$\frac{f(x_{1},\ldots ,x_{n})}{g(x_{1},\ldots ,x_{n})}=\prod_{k=1}^{n}\frac{p_{k}\left(x_{k}\right)}{p_{k-1}\left(x_{k}\right)},$$

as $g(x_{1},\ldots ,x_{n})$ is easily sampled from the trivial $p_{0}\left(x\right)$.

In practice, one uses $g(x_{1},\ldots ,x_{n})$ to generate $N_{s}$ samples of all the intermediate distributions, such that for every set of values $\left\{x_{1}^{i},x_{2}^{i},\ldots ,x_{n}^{i}\right\}$, with $i=1,2,\ldots ,N_{s}$, one obtains a set of weights $\left\{\omega_{i}\right\}$ upon substitution in Equation (8). In this way, 〈α〉 is estimated according to

(9) $$\langle \alpha \rangle \approx \frac{\sum_{i=1}^{N_{s}}\omega_{i}\alpha \left(x_{n}^{i}\right)}{\sum_{i=1}^{N_{s}}\omega_{i}},$$

with

(10) $$\omega_{i}=\prod_{k=1}^{n}\frac{p_{k}\left(x_{k}^{i}\right)}{p_{k-1}\left(x_{k}^{i}\right)}=\frac{Z_{0}}{Z_{n}}\prod_{k=1}^{n}\frac{{\tilde{p}}_{k}\left(x_{k}^{i}\right)}{{\tilde{p}}_{k-1}\left(x_{k}^{i}\right)}=\frac{Z_{0}}{Z_{n}}{\tilde{\omega }}_{i},$$

which defines the set of importance weights $\left\{{\tilde{\omega }}_{i}\right\}$ obtained from the product of the ratios of the unnormalized probabilities. Notice that ${\tilde{\omega }}_{i}$ is an accessible quantity, while $\omega_{i}$ is not, just because one does not have access to $Z_{n}$. One important consequence of this formalism is that a simple estimator of the partition function $Z_{n}$ associated to the distribution $p_{n}\left(x\right)=p\left(x\right)$ is directly given by the average value

(11) $$\frac{Z_{n}}{Z_{0}}\approx \frac{1}{N_{s}}\sum_{i}{\tilde{\omega }}_{i}.$$

Since the values of ${\tilde{\omega }}_{i}$ are usually large, one typically draws samples of $\log({\tilde{\omega }}_{i})$. In this way, one uses a set of $Z_{0}$-normalized AIS samples $s_{i}=\log\left({\tilde{\omega }}_{i}\right)+\log\left(Z_{0}\right)$, such that

(12) $$\log\left(Z_{n}\right)\approx \log\left[\frac{1}{N_{s}}\sum_{i}e^{s_{i}}\right]=\log\left(Z_{\mathrm{AIS}}\right),$$

and defines $Z_{\mathrm{AIS}}$ as an approximation to $Z_{n}$. Notice that this value is different from the mean of the samples $s_{i}$, although these two quantities do not differ much when the variance of the samples is small. In fact, these two quantities tend to be the same when the variance of the set of samples is small compared to the mean value. In other situations, the nonlinear character of the operation in Equation (12) makes the result dominated by the largest samples, to the point that, in the extreme case, the largest sample exhausts the total sum.

## 3. The Restricted Boltzmann Machine

An RBM with binary units is a spin model describing a mixture of two different species, where intra-species interactions are forbidden, and units play the role of the spins. In general, though, RBM units take [0,1] values rather than [−1,1]. Furthermore, only one component of this mixture is assumed to be accessible to the external observer, usually called the *visible layer*. The other species, usually called the *hidden layer*, is assumed to have no contact with the outside world, and is present to build up correlations in the model. As a consequence, one is only interested in the marginal probability distribution associated to the visible units.

The energy function of a binary RBM with $N_{v}$ visible units x and $N_{h}$ hidden units h is defined as [28,29]:

(13) $$E\left(x,h\right)=-x^{T}b-c^{T}h-x^{T}Wh,$$

where W is the two-body weight matrix setting the coupling strength between the two species, while b and c represent the external fields acting on each layer and are generically denoted as *bias*. In this expression, $x^{T}$ stands for the transpose of vector x.

The energy in Equation (13) can be cast as a quadratic form, where visible and hidden units are organized as row and column vectors preceded by a constant value of 1 to account for the bias terms

(14) $${\tilde{x}}^{T}=\left(1\;x_{1}\;x_{2}\cdots x_{N_{v}}\right)\;\;\;\;\;,\;\;\;\;\;{\tilde{h}}^{T}=\left(1\;h_{1}\;h_{2}\cdots h_{N_{h}}\right),$$

leading to

(15) $$E\left(\tilde{x},\tilde{h}\right)=-{\tilde{x}}^{T}\left(\begin{array}{cc} 0 & c^{T}\\ b & W \end{array}\right)\tilde{h}\equiv -{\tilde{x}}^{T}\tilde{W}\tilde{h},$$

where $\tilde{W}$ is the *extended* weight matrix, which includes the bias terms.

As usual in energy-based models, the probability of a state (x,h) follows a Boltzmann distribution

(16) $$p\left(x,h\right)=\frac{e^{-E(x,h)/T}}{Z},$$

with

(17) $$Z=\sum_{x,h}e^{-E(x,h)/T}$$

and $k_{B}$ set to 1. The particular form of the energy function (13) makes both P(h|x) and P(x|h) factorize as a product of probabilities corresponding to independent random variables. As a consequence, Gibbs sampling can be efficiently used to compute them [30]. In addition, it is also possible to evaluate one of the two sums involved in the partition function. In this way, for [0,1] units, one has

(18) $$Z=\sum_{x}e^{x^{T}b/T}\prod_{j}\left(1+e^{(c_{j}+x^{T}W_{\;j})/T}\right),$$

where index *j* runs over the whole set of hidden units, and $W_{\;j}$ stands for the *j*th column of W. However, the evaluation of *Z* is still prohibitive when the number of input and hidden variables is large, since it involves an exponentially large number of terms. For that reason, RBMs are computationally hard to evaluate or simulate accurately [31].

## 4. Parameters of the Models

In this work, we explore different problems where log(Z) can be exactly computed, which will be then used to benchmark the approximations described afterwards. At the end, these are employed to predict the value of log(Z) on a large, realistic system where an exact evaluation is prohibitive. The set of models where the exact log(Z) is accessible include artificially generated weights, magnetic spin systems that can be directly mapped into an RBM, and weights obtained after an RBM learning process (where a training dataset is available, in contrast to the other cases). The weights and bias generated have similar statistical moments, so that by changing the temperature, the system displays different thermodynamic properties. In the following, we focus on the low-temperature regime, as in this limit, the number of states that acquire a significant probability is reduced, as a consequence of the third law of thermodynamics. Due to the large size of the configuration space, the problem of finding log(Z) becomes much harder than at high temperatures, thus challenging the accuracy of the AIS predictions obtained with a low computational cost.

The sets of parameters analyzed in this work include:

(1) Gaussian Weights with Gaussian Moments (GWGM), characterized by an extended matrix of weights $\tilde{W}$ of Gaussian random numbers with $N_{v}=20$ and $N_{h}=180$.
  We have generated a total of 100 models, each one with weights and bias sampled from a normal distribution N(μ,σ), with both μ and σ also sampled from normal distributions. In particular, μ is drawn from N(−10,10) and σ from N(20,10), ensuring the latter is positive. In this way, each model follows a single Gaussian mode with different mean and variance. Notice that there is no explicit temperature dependence in these models, although according to the definition of the RBM energy in Equation (13), a temperature *T* in the corresponding Boltzmann factors could be understood as being reabsorbed into the weights and bias themselves. Finally, due to the reduced value of $N_{v}$, the exact value of *Z* for each model has been calculated by brute force.
(2) A set of weights obtained after training an RBM with the MNIST dataset [32], with $N_{h}=20$ hidden units (MNIST-20h), similar to the simple case studied in Ref. [13]. The network was trained with CD_1_ for 500 epochs where convergence was already achieved. We monitor and store the weights along the learning process with the aim of having a complete picture of their evolution. In this way, we have snapshots taken at the beginning of the learning, where the training set typically does not correspond to the highest probability states, and at the end, where they are supposed to carry most of the probability mass. Notice that this, together with the MNIST-500h model described at the end of this section, are the only problems where standard RBM learning has been performed. Furthermore, being a learning problem, there is no explicit temperature implied, or equivalently, the temperature is always set to 1.

**Figure 1:**
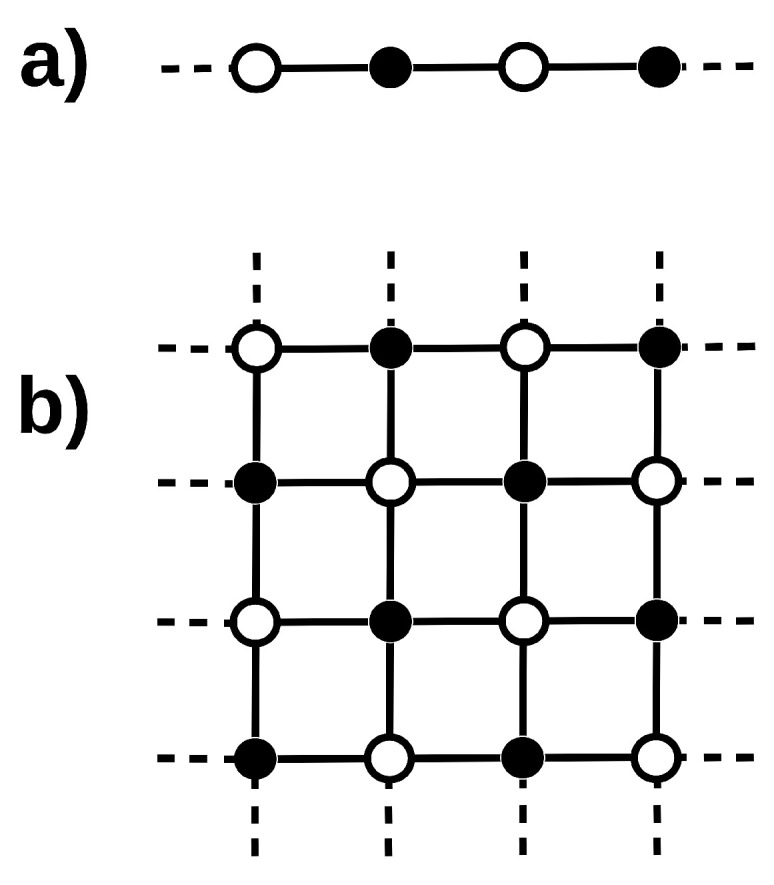
Examples of checkerboard configurations representing 1D (**a**) and 2D (**b**) magnetic spin systems. Black and white circles correspond to visible and hidden units, when mapped into RBMs.

The previous problems use [0,1] binary visible and hidden variables. The next two models correspond to magnetic spin systems, mapped into RBMs using [−1,+1] values, which has been an active topic of research in recent years [33,34,35,36,37]. According to [15,16,17], spin systems with nearest-neighbor interactions can be simulated considering two disjoint subnets with half the total number of spins each. In this scheme, the state of all the spins can be updated in parallel in each subnet. This is a perfect fit for an RBM implementation, where units in the visible and hidden layers are arranged according to a checkerboard configuration, as shown in Figure 1. Actually, using an RBM with these weights yields an exact mapping to the standard procedure of sampling the two disjount networks mentioned above.

(3) Classical Ising and Spin Glass models in one and two dimensions. A one-dimensional Ising model with periodic boundary conditions containing an even number of spins $\left\{s_{1},s_{2},\ldots ,s_{2N}\right\}$ can be represented by an RBM with the same number of units in each layer, as shown in panel (a) of Figure 1. Identifying even and odd spins with hidden and visible units, corresponding to black and white symbols in the figure, one has
  $$\begin{array}{ccc} b^{T}\;\;\; & = & \;\;\;(B_{1},B_{3},\cdots ,B_{2N-1})\\ c^{T}\;\;\; & = & \;\;\;(B_{2},B_{4},\cdots ,B_{2N}) \end{array}$$
  and
  $$\begin{array}{ccc} W\;\;\; & = & \;\;\;\left(\begin{array}{ccccc} J_{1,2} & 0 & 0 & \cdots & J_{N,1}\\ J_{2,3} & J_{3,4} & 0 & \cdots & 0\\ 0 & J_{4,5} & J_{5,6} & \cdots & 0\\ \vdots & \vdots & \vdots & \ddots & \vdots \\ 0 & 0 & 0 & \cdots & J_{N-1,N} \end{array}\;\;\right), \end{array}$$
  where $J_{i,i+1}$ is the interaction between spins $s_{i}$ and $s_{i+1}$. Only two entries per row/column can be non-zero in this arrangement. In the Ising model (1DIsing), $J_{i,i+1}=J$ and $B_{i}=B$ for all spins, while they can take different values in what we denote as a Spin Glass model (1DSG). The partition function of 1DIsing and 1DSpinGlass can be easily computed using the Transfer Matrix formalism [38,39]. We have generated 100 different 1DIsing models, with the J and B parameters drawn from a normal distribution with μ=−1 and σ=2. That gives 100 different 1DIsing Hamiltonians. In much the same way, we have also generated 100 1D Spin Glass models, with all the $J_{i,j}$ and $B_{j}$ parameters drawn from the same probability distribution. All these models contain $N_{s}=200$ spins. We have then analyzed these systems at three different temperatures, T=0.1 (1DIsing1 and 1DSG1), T=0.01 (1DIsing2 and 1DSG2), and T=0.001 (1DIsing3 and 1DSG3).
  The two-dimensional square-lattice Ising model is much harder to solve and its analytic solution was given by Onsager in [40] in the absence of an external field. Similar to the 1D models, it can be represented by an RBM, where visible and hidden units are arranged in a checkerboard configuration, as shown in panel (b) of Figure 1. In this case, four weights can be non-zero in each row and column of $\tilde{W}$ since there are no bias terms. Three sets of 100 2DIsing models (2DIsing1, 2DIsing2 and 2DIsing3) corresponding to $N_{s}=256$ spins have been generated, with parameters drawn from the same normal distributions used for the previous 1D cases, and the same temperatures.
  Furthermore, we have extended that to what we call a 2D Spin Glass (2DSG), where all two-body $J_{i,j}$ correlations are different, while keeping the connectivity restricted to nearest neighbors. In this case, the partition function is computed by *brute force*, which limits the size of the square lattices to less than or equal to 6×6, as an even number of spins per dimension is required in order to properly satisfy the periodic boundary conditions. Two different sets of 50 models (2DSG1 and 2DSG2) have been used, drawn from a normal distribution with μ=1 and σ=1 and corresponding to $T=10^{-3}$ and $T=10^{-5}$, respectively.

All these models use [−1,1] spin variables as standard.

Finally, we also analyze the weights of an RBM trained with the MNIST dataset containing $N_{h}=500$ hidden units (MNIST-500h), where no exact value of log(Z) can be obtained due to its large size. The training was made in the same conditions as in the MNIST-20h case.

## 5. The Optimal Mean Field Approximation

The equilibrium Boltzmann distribution associated to any physical system is given by

(19) $$p\left(x\right)=\frac{e^{-E(x)/T}}{Z},$$

where E(x) is the system’s energy corresponding to state x. In the spirit of AIS, the partition function associated to $p_{n}\left(x\right)$ can be obtained from a chain of intermediate probability distributions that start from another, much simpler and easy-to-sample $p_{0}\left(x\right)$, as shown in Section 2. Obtaining a good $p_{0}\left(x\right)$ can ease the job for AIS, and therefore becomes a key ingredient to obtain an accurate estimation of log(Z) with a reasonable number of intermediate chains and samples. A very simple probability distribution $p_{0}\left(x\right)$ can be obtained from a mean-field model containing only external fields B. In this scheme, and for an RBM, $E_{0}\left(x\right)=-x^{T}B$ defines the starting mean field energy, which makes

(20) $$p_{0}\left(x\right)=\frac{2^{N_{h}}e^{x^{T}\cdot B/T}}{Z_{0}}$$

the product of independent distributions for each unit, thus allowing for a very simple and efficient sampling scheme in parallel. Furthermore, for [0,1] binary units, the corresponding partition function reads

(21) $$Z_{0}=2^{N_{h}}\prod_{j=1}^{N_{v}}\left(1+e^{B_{j}/T}\right)$$

while for [−1,1] units, one has

(22) $$Z_{0}=2^{N_{v}+N_{h}}\prod_{j=1}^{N_{v}}\cosh\left(B_{j}/T\right)\;.$$

Despite dealing with a mean field, obtaining the most suitable B may not be a trivial task. In most practical applications, and for lack of a better model, the simplest choice B=0 is adopted, thus turning $p_{0}\left(x\right)$ into the uniform probability distribution. In the spirit of the AIS algorithm, and according to the theoretical development [9,10], one then expects that increasing the number of intermediate distributions should lead to the exact result, no matter what the starting $p_{0}\left(x\right)$ is. Whilst this should be the case, the dynamics of this process are not clear, nor is it clear whether the desired limit is attained with a large but manageable number of intermediate distributions. In other words, one has no clue as to what the convergence properties of the algorithm are, other than knowing that it provides the right result in the infinite limit. In order to test that in practice, we have conducted different experiments with the GWGM and MNIST-20h problems of Section 4. Given that our goal is to obtain reliable estimates of log(Z) with a small computational cost, these experiments are also useful for selecting suitable values for $N_{\beta }$ and $N_{s}$. In these experiments and the following ones reported, a linear grid of equidistant inverse temperatures has been employed. We have tried different schemes (such as a logarithmic grid), to find that no significant differences were obtained.

**Figure 2:**
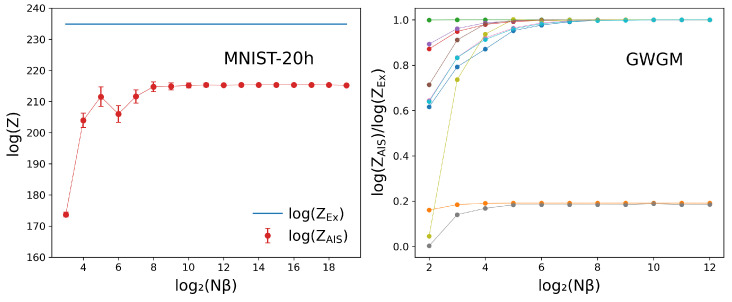
AIS estimation of log(Z) starting from B=0 for the MNIST-20h (left) and ten different GWGM sets of weights (right) as a function of the number $N_{\beta }$ of intermediate distributions. The left panel shows both the exact value (in blue) and the AIS estimations, while on the right, the ratio of these two quantities is plotted.

Figure 2 shows the evolution of the prediction of log(Z) with $N_{\beta }$ for the MNIST-20h (left panel) and 10 randomly selected GWGM weights (right panel). In all these calculations, a total of $N_{s}=1024$ AIS samples have been employed to build $\log(Z_{AIS})$ according to Equation (12). In the MNIST-20h case, both the exact and the predicted values are displayed, while in the GWGM case, the ratio of the AIS log(Z) to the exact log(Z) is displayed for the sake of clarity. The error bars are obtained after averaging 100 repetitions of the same experiments.

Two immediate conclusions can be drawn from Figure 2. On one hand, it is clear that in both cases, a stable prediction has been achieved already at $N_{\beta }=N_{s}=1024$. This fact has also been observed with all the sets of weights tested. Starting from there, we have set $N_{\beta }=4096$ and $N_{s}=1024$ in all the following AIS runs throughout this work, which seems to be large enough to obtain stable results while still allowing for a fast evaluation of log(Z) with a standard computer. On the other hand, one readily notices that, despite providing an apparently converged result, the AIS prediction starting from B=0 may differ substantially from the exact result, even in cases where one of the dimensions of the problem ($N_{v}$ or $N_{h}$) is small. The situation is even worse as the error bars diminish with increasing $N_{\beta }$, leading to the false impression that a reliable prediction has been achieved. The results in the left panel show that this picture remains unaltered even with $N_{\beta }=2^{20}$, thus indicating that a completely unpractical amount of intermediate distributions is probably needed to produce the required changes to bring the AIS prediction close to the exact result, something that is guaranteed in the asymptotic limit [9,10].

Still, the plots in Figure 2 yield a discouraging picture about the possibility of achieving good results starting from B=0, an image that should be properly put into perspective. In order to obtain a more complete view, we have conducted AIS experiments starting from B=0 on all the models described in Section 4.

**Figure 3:**
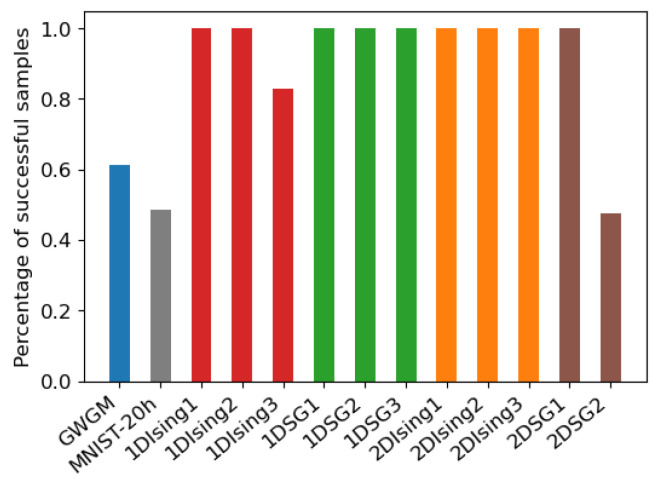
Percentage of AIS samples producing an estimation of log(Z) with a relative error of less that 5% with respect to the exact result, obtained starting from B=0. The results have been averaged over all sets of weights corresponding to the same problem.

We have computed 10 independent repetitions for each set of weights, each consisting of $N_{s}=1024$ AIS samples with $N_{\beta }=4096$. For every repetition, an estimation of log(Z) has been obtained from the 1024 samples using Equation (12), and the relative error

(23) $$\epsilon_{r}=\left|\frac{\log\left(Z_{\mathrm{Ex}}\right)-\log\left(Z_{\mathrm{AIS}}\right)}{\log(Z_{\mathrm{Ex}})}\right|$$

has been calculated. For all the set of weights belonging to the same system (GWGM, MNIST-20h, …), the total number of estimations producing a relative error $\epsilon_{r}\leq \;0.05$ have been computed. The bars in Figure 3 show that number as a percentage. As can be seen, the choice B=0 works in many cases, but not in all of them.

In any case, and despite the fact that the uniform probability distribution corresponding to B=0 provides a trivial starting point, it is not the only possible simple choice. In fact, any distribution of the mean field form given in Equation (20) is suitable to start AIS from, as all components of x become independent random variables that can be sampled in parallel. Among all the possible choices of B, therefore, one can look for the optimal one that produces the best possible results with little computational cost. In this context, being optimal means producing a mean field probability distribution that is closest to the actual $p_{n}\left(x\right)$ one seeks to sample, according to some metric.

In particular, the optimal values $B^{*}$ of B can be obtained minimizing the Kullback–Leibler (KL) divergence between $p_{0}\left(x\right)$ and the full RBM probability distribution $p_{n}\left(x\right)$, so we impose the condition

$${\left.\nabla_{B}\sum_{x}p_{n}\left(x\right)\log\left(\frac{p_{n}\left(x\right)}{p_{0}\left(x\right)}\right)\right|}_{B=B^{*}}=0\;,$$

where the sum over x extends to all the $2^{Nv}$ states, as hidden states have already been marginalized in both $p_{0}\left(x\right)$ and $p_{n}\left(x\right)$. One thus has, for $x_{i}\in \left[0,1\right]$,

(24) $$\begin{array}{ccc} 0 & = & {\left.-\sum_{x}p_{n}\left(x\right)\nabla_{B}\log p_{0}\left(x\right)\right|}_{B=B^{*}}\\ & = & -\frac{1}{T}\sum_{x}p_{n}\left(x\right)x+{\left.\sum_{x}p_{n}\left(x\right)\nabla_{B}\log Z_{0}\right|}_{B=B^{*}}\\ & = & -{\langle x\rangle }_{n}+\frac{1}{1+e^{-B^{*}/T}}\;, \end{array}$$

where the subscript *n* indicates that the average values are taken over the $p_{n}\left(x\right)$ probability distribution corresponding to the target RBM. In this way, one obtains, for $x_{i}\in \left[0,1\right]$,

(25) $$B^{*}=-T\log\left(\frac{1}{{\langle x\rangle }_{n}}-1\right)$$

for each visible unit $i\in \{1,2,\ldots ,N_{v}\}$. For $x_{i}\in \left[-1,1\right]$, a similar procedure leads to

(26) $$B^{*}=-T\tanh^{-1}\left({\langle x\rangle }_{n}\right)\;.$$

These expressions, also appearing in [41], imply that the problem of finding $B^{*}$ is equivalent to obtaining the exact average values of the visible units, which may not be a trivial task depending on the problem at hand.

**Figure 4:**
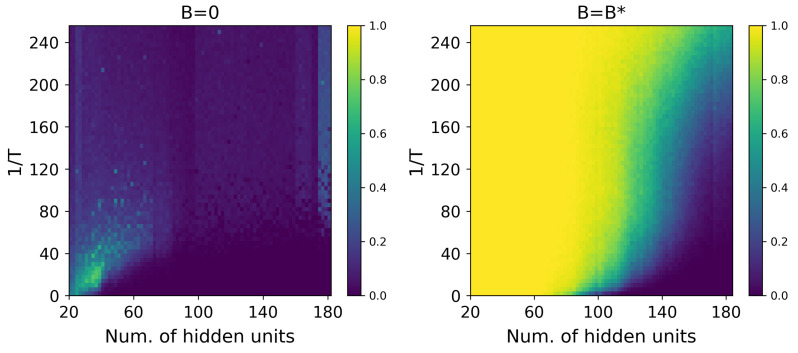
Percentage of AIS samples producing a relative error lower than or equal to 5% with respect to the exact log(Z) value, as a function of the number of hidden units and inverse temperature for a representative GWGM model. The left and right panels show the results starting from B=0 and $B=B^{*}$, respectively.

In order to test the benefits of using $B^{*}$, we perform several AIS runs starting from the optimal $p_{0}^{*}\left(x\right)=2^{N_{h}}e^{B^{*}x/T}/Z_{0}$ and compare the results to the same calculations starting from the uniform probability distribution, corresponding to B=0. As stated above, in both cases, we use $N_{\beta }=4096$ intermediate chains to obtain $N_{s}=1024$ AIS samples. Figure 4 shows the results obtained in colormap form for one of the most difficult GWGM cases. The horizontal axis indicates the number $n_{h}$ of hidden units considered, spanning the range from 1 to $N_{h}=180$, obtained by discarding weights (that is, setting $\omega_{ij}=0$ for $j>n_{h}$), while the vertical axis displays the inverse temperature. In all cases, we use $N_{v}=20$ visible units, as described in Section 4, thus allowing for the exact calculation of log(Z) by *brute force*. The maps show the percentage of the 1024 samples of log(Z) that differ from the exact value by less than 5% in each case. As can be readily seen, the fact that $p_{0}^{*}\left(x\right)$ is *closer* to the RBM probability distribution makes AIS work less and perform better, as expected. Notice, though, that for some combinations of *T* and $n_{h}$, the efficiency of AIS suffers even when starting from $p_{0}^{*}\left(x\right)$. This should not be completely surprising, mostly considering that a mean field starting probability distribution can still be too far away from that of the actual RBM, thus indicating that one should look for a different (and unknown) starting probability distribution.

**Figure 5:**
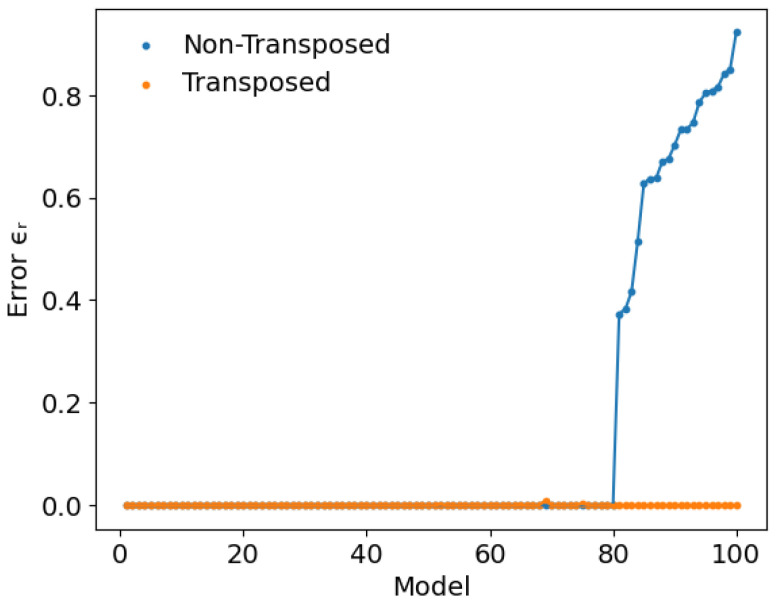
Relative error of all models in the transposed and non-transposed GWGM weights, computed as in Equation (23). For the sake of clarity, the models have been sorted according to the relative error of the non-transposed results.

The right panel in Figure 4 also suggests that a mean field starting point can be problematic when the number of hidden units is much larger than the number of visible ones. This problem is easily solved noticing that log(Z) is invariant under the exchange of x and h in the RBM, associated to replacing the array of weights by its transpose. Based on these results, we have conducted additional tests on the whole GWGM set. In fact, the expectation values ${\langle x\rangle }_{n}$ can always be evaluated when the dimension of the hidden space is small, as in the present case. It is easy to show that, for binary [0,1] units, one has

(27) $${\langle x\rangle }_{n}=\sum_{h}p_{n}\left(h\right)\prod_{i=1}^{N_{v}}\frac{1}{1+e^{-(b_{i}+W^{i}h)/T}},$$

where the sum extends over all hidden states, while $p_{n}\left(h\right)$ and $W^{i}$ stand for the hidden state probability and the i-th row of the two-body weight matrix, respectively. Figure 5 shows the relative error obtained after averaging ten repetitions of each AIS run, for the 100 GWGM models. All runs started from $B^{*}$, computed from the exact ${\langle x\rangle }_{n}$, for the transposed and non-transposed configurations. Results have been sorted in ascending error order of the non-transposed configurations in order to obtain a better view. As can be seen, all models are accurately reproduced in the transposed case, where the number of hidden units is smaller than the number of visible ones. On the contrary, about 20% of the models show large deviations from the exact result when the original, non-transposed model is evaluated. This behavior is also observed when performing similar calculations with the other problems presenting large differences in the number of hidden and visible units.

## 6. Approaching the Optimal Mean Field

Despite the simplicity of the expressions in Equations (25) and (26), the problem of finding the optimal $B^{*}$ can actually be as hard as finding log(Z) itself, so one has to devise alternative strategies to approximate it.

Three common strategies are usually employed to face this problem [14]. The simplest one is to disregard Equations (25) and (26), set B=0 and sample from the uniform probability distribution, as discussed above. Another common strategy is to set B=b from Equation (13) and to disregard the contributions of the hidden units. Despite its simplicity, the resulting $p_{0}\left(x\right)$ is usually far away from $p_{n}\left(x\right)$. The third approach was devised in [13] for the specific case of RBM learning, where ${\langle x\rangle }_{n}$ is approximated by its average over the training set. However, this procedure cannot be employed when a training set is lacking, as when dealing with magnetic spin systems for instance, or when the existing training set does not properly represent the underlying probability distribution of the system.

In this work, we introduce two alternative strategies to estimate $B^{*}$ that, on the one hand, imply a low computational cost, and on the other, avoid some of the drawbacks of the aforementioned choices. They both rely on finding a suitable approximation to compute ${\langle x\rangle }_{n}$ in Equations (25) and (26). At this point, many different choices are possible, while keeping in mind that none of them will perfectly reproduce the exact ${\langle x\rangle }_{n}$, as we assume the original $p_{n}\left(x\right)$ is intractable. However, one must keep in mind that the resulting probability distribution obtained from them is used as the initial point for AIS, which will afterwards correct that to produce reliable samples of $p_{n}\left(x\right)$.

Among the many possible choices, we introduce the following ones:

- Pseudoinverse (Pinv) approximation: One can look for a state of the complete (visible and hidden) space with large probability. In this case, one works directly with the energy, setting to zero the gradients with respect to x of the expression in Equation (13). One then finds
  (28) $$x_{p}=-{\left(W^{+}\right)}^{T}c$$
  where $W^{+}$ is the pseudoinverse of the W matrix. In this work, we build $x_{p}$ by rounding the result of Equation (28) to the [0,1] or the [−1,1] range, depending on the units used, and approximate ${\langle x\rangle }_{n}$ by $x_{p}$. With that, we build the corresponding mean-field bias $B_{\mathrm{Pinv}}$.
- Signs from Random Hidden (Signs_h): The expectation values ${\langle x\rangle }_{n}$ given in Equation (27) can only be evaluated when the number of hidden units is small, but unfortunately, that is not usually the case in real problems. For that reason, we resort to a heuristic approximation, where a set of hidden states $h^{\left(\alpha \right)}$ randomly selected from the uniform probability distribution is used to obtain the same number of visible states $x^{\left(\alpha \right)}$ from the conditional probabilities $p\left(x_{i}^{\left(\alpha \right)}=1|h_{\alpha }\right)=1/\left(1+e^{-(b_{i}+W^{i}h^{\left(\alpha \right)})}\right)$. This expression assigns a probability larger than 0.5 to $x_{i}^{\left(\alpha \right)}=1$ depending on the sign of the argument in the exponential. Following this, we set the components of $x^{\left(\alpha \right)}$ to be equal to 1 when $b_{i}+W^{i}h^{\left(\alpha \right)}>0$, and to 0 in the opposite case. As in most of the calculations performed in this work, we build a set of 1024 uniformly sampled $h^{\left(\alpha \right)}$ that are used to generate the $x^{\left(\alpha \right)}$, which are finally averaged to obtain the estimation of ${\langle x\rangle }_{n}$ required to compute the approximated bias $B_{\mathrm{Signs}\_h}$. Notice that this is a cost-effective procedure that involves less operations than the pseudoinverse procedure outlined above. This approach is trivially extended to [−1,1] units.

**Figure 6:**
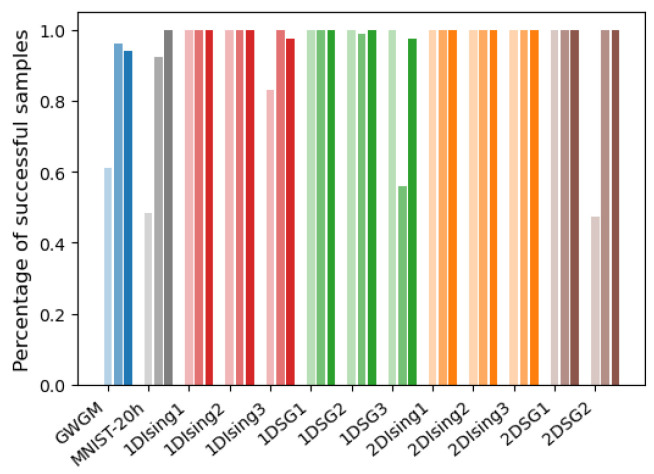
Percentage of AIS samples with a relative error lower than 0.05% with respect to the exact log(Z) for the different problems analyzed. The left, middle and right bars with different gray levels correspond to the predictions starting from B=0, $B=B_{\mathrm{Pinv}}$ and $B=B_{\mathrm{Signs}\_h}$, respectively.

These two strategies have been used to produce the mean-field probability distributions of Equation (20) that are used to start AIS. We perform 10 repetitions of each experiment for each model, producing a total of 1000 final values for the GWGM weights. Figure 6 shows the statistics obtained for all the cases analyzed, corresponding to the total amount of AIS predictions producing a relative error of less than 5% with respect to the exact value of log(Z). The lighter, midtone and darker bars correspond to B=0, $B=B_{\mathrm{Pinv}}$ and $B=B_{\mathrm{Signs}\_h}$, respectively. As can be seen, both Pinv and Signs_h outperform B=0 in most cases, yielding similar results in general. It is also worth noticing that for the sets that do not have bias (b=c=0 in Equation (13)), B=0 is the optimal $B^{*}$ when [−1,1] units are employed. In this case, all three strategies yield very good and similar results.

**Figure 7:**
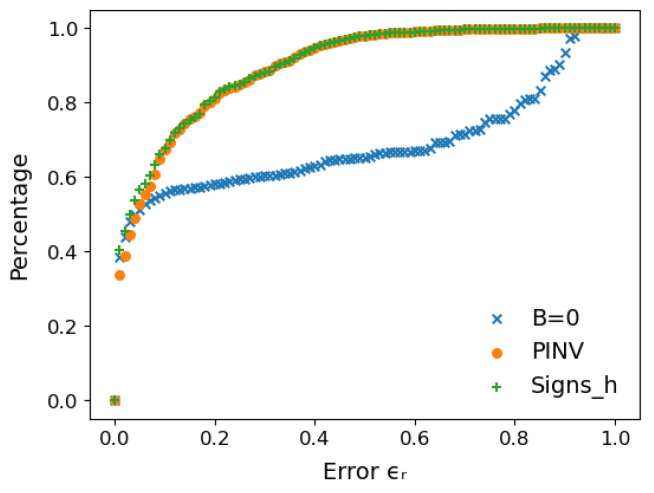
Percentage of GWGM AIS samples with a relative error lower than or equal to $\epsilon_{r}$ with respect to the exact log(Z).

The fact that both $B_{\mathrm{Signs}\_h}$ and $B_{\mathrm{Pinv}}$ lead to overall better AIS predictions than B=0 is a direct consequence of the distribution of AIS samples in each case. This is illustrated in Figure 7 for the GWGM case, where all samples generated from all repetitions of all models have been used to account for better statistics. The plot shows the percentage of samples that have a relative error with respect to the exact log(Z) equal to or lower than $\epsilon_{r}$, as a function of $\epsilon_{r}$, for the B=0, $B=B_{\mathrm{Signs}\_h}$ and $B=B_{\mathrm{Pinv}}$ strategies. As can be seen, the B=0 mean field performs worse than the other two in general, although all three strategies produce similar results up to ϵ≈0.05. For higher values, though, differences are significant, converging once again towards the end of the curve where all samples fulfill the condition. In any case, we find that $B=B_{\mathrm{Signs}\_h}$ and $B=B_{\mathrm{Pinv}}$ perform very similarly, with minor variations that in the end lead to the small prediction differences displayed in Figure 6. One can thus conclude that, overall, the samples generated by $B=B_{\mathrm{Signs}\_h}$ and $B=B_{\mathrm{Pinv}}$ are closer to the exact value of log(Z) than the set produced by B=0. Despite that, one could argue that in all cases, there is always a large amount of samples that fail to predict anything close to the right value. However, it is worth noticing that this should be the case due to the stochastic nature of the AIS algorithm and the exponential way in which the generated samples have to be combined, as displayed in Equation (12). Fluctuations above the exact value of log(Z) are exponentially amplified, and have to be compensated by a large amount of samples that underestimate its value, whose contribution is exponentially diminished. We can thus conclude that the AIS algorithm has to produce a lot of apparently bad samples in order to produce an accurate result. Furthermore, this asymmetric generation of samples above and below the exact value leads, when not properly balanced, to an underestimation of log(Z), as noticed in [42]. This picture, though, can be alleviated by increasing the number of intermediate chains $N_{\beta }$, at the expense of linearly increasing the computational cost.

**Figure 8:**
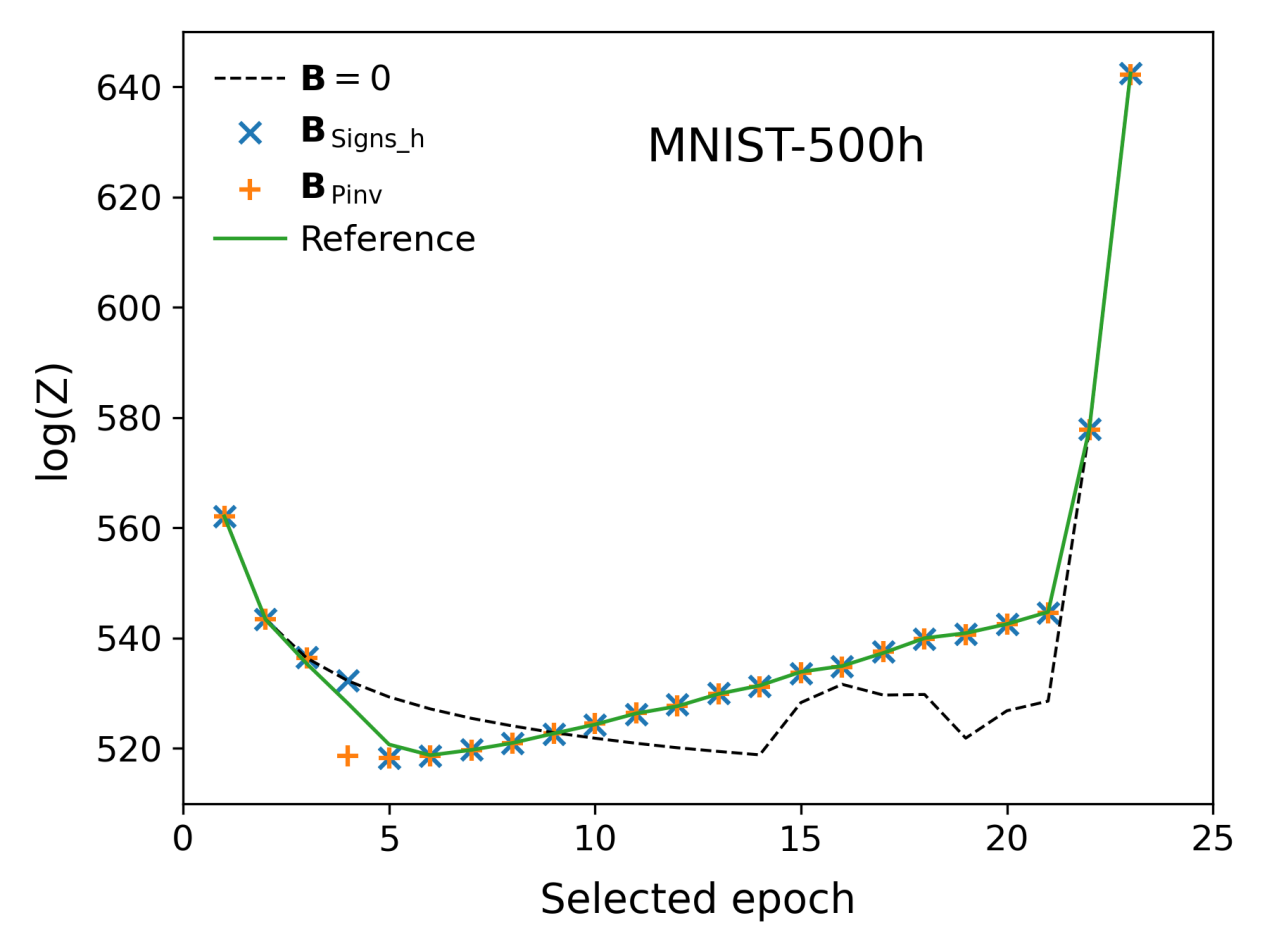
Comparison of the AIS estimation of log(Z) along learning for the MNIST dataset with 500 hidden units obtained starting from the different mean field probability distributions discussed in this work. The first points correspond to the first epochs, while the last ones show the predictions obtained at an intermediate stage.

We finally close the discussion by showing in Figure 8 the value of the partition function estimated with AIS for the MNIST dataset, using an RBM model containing $N_{h}=500$ hidden units.

For this system, due to its large size, there is no exact calculation of log(Z) and one has to rely on the predictions obtained employing state-of-the-art techniques found in the literature. For that matter, we take as reference the value obtained from the procedure outlined in Ref. [13], where the dataset used to train the RBM is also employed to approximate the mean values required for the evaluation of $B^{*}$ in Equations (25) and (26). With this, we run AIS with $N_{s}=1024$ and $N_{\beta }=2^{20}$ to obtain the reference value (green solid line in the figure). Notice that $N_{\beta }$ is unreasonably large compared to what one would normally use in order to obtain a maximally accurate approximation of log(Z) with the same number of samples used throughout this work. The figure also shows the estimations obtained using B=0, $B=B_{\mathrm{Signs}\_h}$ and $B=B_{\mathrm{Pinv}}$ (dotted line, crosses and plus symbols, respectively). The first 21 points correspond to the first 21 epochs where the RBM weights rapidly evolve, while the last two points correspond to epochs 40 and 100. As can be seen, all curves merge at the highest epochs, while the B=0 prediction departs from the reference curve at the early and intermediate epochs. On the contrary, the selected strategies are hardly distinguishable from the reference line along the whole curve. Despite the fact that the differences between the B=0 curve and the rest are small, one should realize that the computational cost involved in using the proposed strategies is very low, while the predictions obtained are closer to the reference value. This is something that should be taken into account if the goal is to obtain the most accurate but economic prediction of log(Z).

## 7. Summary and Conclusions

To summarize, we have analyzed the performance of the AIS algorithm with a reduced number of samples and intermediate chains in the evaluation of the partition function *Z* of magnetic spin and spin-like systems using Restricted Boltzmann Machines. We estimate log(Z) for a number of exactly solvable models where the exact value of log(Z) is known. In particular, we show that a suitable starting probability distribution $p_{0}\left(x\right)$ of the mean field form can lead to a big improvement of the AIS estimation of log(Z) for a fixed number of samples and intermediate chains. In this scheme, we build $p_{0}\left(x\right)$ from the optimal mean-field approximation that minimizes the Kullback–Leibler distance to the probability distribution implemented by the RBM describing the system. These mean fields are directly related to the statistical average of the spin configurations. Remarkably and despite using an RBM to describe the system, our methodology does not require a training set, and thus, it can be used when none are available. The procedure requires only sampling the RBM.

We also propose two simple strategies to approximate the optimal mean field for large systems where the exact averages cannot be computed. These result from a trade-off between simplicity, reduced computational cost, and accuracy. The first strategy requires the pseudo-inversion of the matrix of RBM weights, while the second is much cheaper and involves only checking the signs of a linear transformation of it. Overall, both strategies perform equal to or better than the standard procedure that starts from B=0 in the cases analyzed, where log(Z) is directly accessible. We have tested them on the MNIST dataset with 500 hidden units, to show that the estimations obtained are in excellent agreement with the ones from the procedure outlined in Ref. [13]. The methods described, though, require a cautious estimation of the average value of the visible units, which may not be easy to obtain. Furthermore, and as discussed in the text, a minimum number of intermediate AIS temperatures and samples is required. Finally, the reader should note that one should try both strategies to obtain the best result.

We expect that the strategies proposed can be used as the starting point in further studies of log(Z) with the AIS algorithm, either in isolated form or combined. Furthermore, since AIS is a general-purpose Importance Sampling algorithm and the proposed strategies are not specific to the computation of log(Z), we expect them to be useful when estimating the expected value of any observable with AIS, for any system that can be mapped into an RBM.

## Data Availability

The raw data supporting the conclusions of this article will be made available by the authors on request.
